# Supplementary figures and images for: Prognostic impact of suspicious extraabdominal lymph nodes on patient survival in advanced ovarian cancer
Source: PLoS One. 2024 May 28;19(5):e0299205. doi: 10.1371/journal.pone.0299205 (PMC11132458; doi:10.1371/journal.pone.0299205)

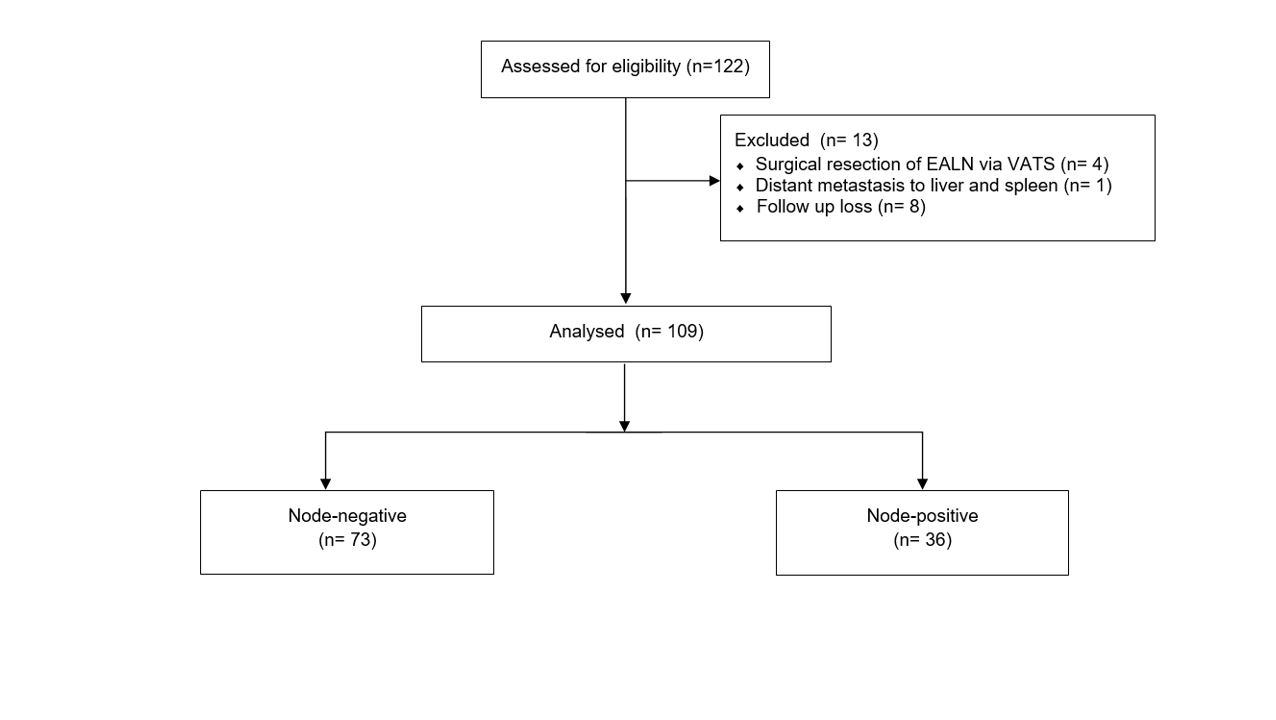

Supplement: S1 Fig — (TIF) [file pone.0299205.s001.tif]

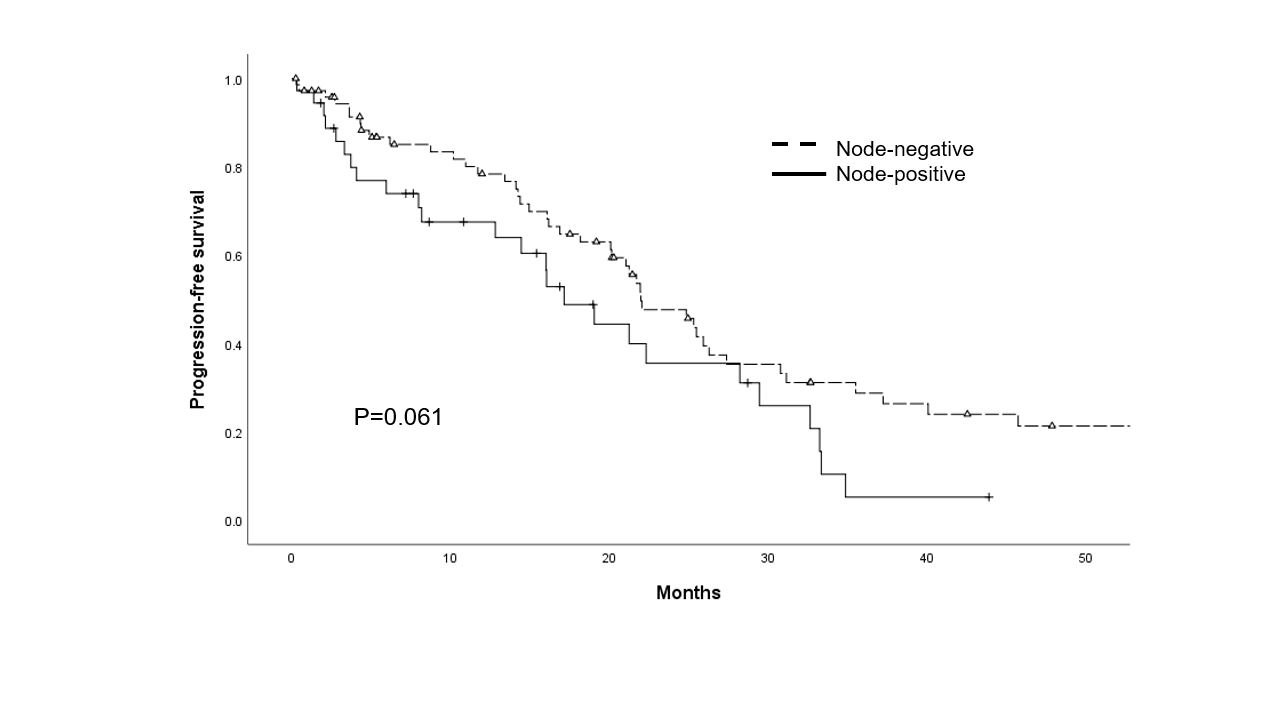

Supplement: S2 Fig — (TIF) [file pone.0299205.s002.tif]
