# Supplementary material for: Prognostic impact of suspicious extraabdominal lymph nodes on patient survival in advanced ovarian cancer
Source: PLoS One. 2024 May 28;19(5):e0299205. doi: 10.1371/journal.pone.0299205 (PMC11132458; doi:10.1371/journal.pone.0299205)
Supplement: Attachment — Submitted filename: Response to Reviewers.docx [file pone.0299205.s003.docx]

**Response to Reviewers**

**Editor Comments:**

1. Please ensure that your manuscript meets PLOS ONE's style requirements, including those for file naming. The PLOS ONE style templates can be found at
   https://journals.plos.org/plosone/s/file?id=wjVg/PLOSOne_formatting_sample_main_body.pdf and 
   https://journals.plos.org/plosone/s/file?id=ba62/PLOSOne_formatting_sample_title_authors_affiliations.pdf

**Address:** We have thoroughly rechecked the manuscript, including those for file naming and file format. We have corrected as per Plos template that is provided.

2. We note that the grant information you provided in the ‘Funding Information’ and ‘Financial Disclosure’ sections do not match. When you resubmit, please ensure that you provide the correct grant numbers for the awards you received for your study in the ‘Funding Information’ section.

**Address:** We did not receive any grants or research funds for this study. We mistakenly included funding-related information from a previous study. We have corrected such error and have made sure that our ‘Funding Information’ and ‘Financial Disclosure’ sections match.

3. Thank you for stating the following in the Acknowledgments Section of your manuscript:
   "This study was partially supported by the research fund from the Sudang Foundation." We note that you have provided funding information that is not currently declared in your Funding Statement. However, funding information should not appear in the Acknowledgments section or other areas of your manuscript. We will only publish funding information present in the Funding Statement section of the online submission form. Please remove any funding-related text from the manuscript and let us know how you would like to update your Funding Statement. Currently, your Funding Statement reads as follows: 
   "The author(s) received no specific funding for this work." Please include your amended statements within your cover letter; we will change the online submission form on your behalf.

**Address:** We have removed the Acknowledgements Section and any other funding-related text from the manuscript and would like to update our Funding Statement as follows: “The author(s) received no specific funding for this work”. We have added this information in our cover letter.

4. In your Data Availability statement, you have not specified where the minimal data set underlying the results described in your manuscript can be found. PLOS defines a study's minimal data set as the underlying data used to reach the conclusions drawn in the manuscript and any additional data required to replicate the reported study findings in their entirety. All PLOS journals require that the minimal data set be made fully available. For more information about our data policy, please see http://journals.plos.org/plosone/s/data-availability.

Upon re-submitting your revised manuscript, please upload your study’s minimal underlying data set as either Supporting Information files or to a stable, public repository and include the relevant URLs, DOIs, or accession numbers within your revised cover letter. For a list of acceptable repositories, please see http://journals.plos.org/plosone/s/data-availability#loc-recommended-repositories. Any potentially identifying patient information must be fully anonymized.

Important: If there are ethical or legal restrictions to sharing your data publicly, please explain these restrictions in detail. Please see our guidelines for more information on what we consider unacceptable restrictions to publicly sharing data: http://journals.plos.org/plosone/s/data-availability#loc-unacceptable-data-access-restrictions. Note that it is not acceptable for the authors to be the sole named individuals responsible for ensuring data access.

We will update your Data Availability statement to reflect the information you provide in your cover letter.

**Address:** All dataset files that support the findings of this study are openly available in Figshare (DOI 10.6084/m9.figshare.24720279).

**Additional Editor Comments:**

**Address:** Thank you so much for your valuable comments.

**Reviewer comments**

Reviewer #1: I read with great interest the Manuscript titled " Prognostic impact of suspicious extraabdominal lymph nodes on patient survival in advanced ovarian cancer”, topic interesting enough to attract readers' attention.
Authors should clarify some point and improve the discussion citing relevant and novel key articles about the topic:
- I suggest a round of language revision, in order to correct few typos and improve readability.
- Considering topic and results if this study, I suggest that authors to add a reference to current evidence about the role of lymphadenectomy in advanced ovarian cancer, considering the advantages and limitations of this procedure. I would be glad if the authors discuss this important point, referring to PMID: 32036457.
Considered all this points, I think it could be of interest for the readers and, in my opinion, it deserves the priority to be published after minor revisions

**Address:** Thank you so much for your comments.

- The attached manuscript has been proofread by Editage, a professional English language editing and publication support service. However, we adhered to your suggestion of additional language revision to correct typos and improve readability.

- We wholly agreed with you. We have added the suggested reference [32] to current evidence about the role of lymphadenectomy in advanced ovarian cancer in line 275-277. **“Randomized trials determining the efficacy of systematic intraabdominal lymphadenectomy have reported an association with PFS, while additional studies have reported a higher incidence of postoperative complications without improved oncological outcome [12, 13, 32].”**

Reviewer #2: It is of great significance to assess the prognostic role of extra-abdominal lymph nodes metastasis in advanced ovarian cancer and weight the advantages and disadvantages of EALNs resection.

Major points:

1. The authors categorized lymph nodes as suspicious, a cut-off value of ≥ 5 mm short-axis dimension, according to the guidelines of the European Society of Urogenital Radiology (ESUR) and previous literature. According to Ref17 Forstner R, Sala E, Kinkel K, Spencer JA. ESUR guidelines: ovarian cancer staging and follow-up. Eur Radiol. 2010;20(12):2773-80, the suspicious lymph nodes were with a short-axis diameter of >1 cm, or suspicious clusters of smaller lymph nodes, or cardiophrenic lymph nodes with a short-axis diameter of >5 mm were considered suspicious. A short-axis diameter of >5mm was only involved in the cardiophrenic lymph nodes in this reference. To the best of my knowledge, the 2022 EUSR (Eur Radiol. 2022 May;32(5):3220-3235) defined lymph nodes metastasis not only considering the short-axis dimension, but also the anatomical position. For example, a short-axis diameter of >1 cm for mediastinal lymph nodes, 1.5 cm for inguinal lymph nodes, and >5 mm for internal mammary and supradiaphragmatic lymph nodes. Since this study included patients with not only supradiaphragmatic lymph nodes, different anatomical information should be considered in the diagnostic criteria.

**Address:** We wholly agreed with you. For further clarification, we have added suggested details pertaining the diagnostic criteria according to the 2022 guidelines of ESUR of suspicious EALNs in line 128-133. **“The short-axis dimension and anatomical position of the largest EALN was measured and recorded. According to the guidelines of the European Society of Urogenital Radiology (ESUR) and previous literature, a cut-off value of ≥ 5 mm short-axis dimension for CPLNs, ≥ 1 cm for mediastinal lymph nodes such as supraclavicular, subcarinal, subphrenic and hilar lymph nodes, ≥ 1.5 cm for inguinal lymph nodes, and ≥ 5 mm for internal mammary and supradiaphragmatic lymph nodes was applied to categorize lymph nodes as suspicious [17].”**

We also thoroughly reviewed our data to confirm that our “node-positive” patients indeed adhered to the mentioned criteria. For example, suspicious mediastinal lymph nodes were detected in 15 of the patients. All were categorized as “node-positive” due to 1) isolated mediastinal lymph node enlargement of short-axis dimension of ≥ 1cm, 2) synchronous cardiophrenic lymph node enlargement of short-axis dimension of ≥ 5mm, or 3) positive PET/CT scans.

2. “Lesions with maximum standardized uptake values (SUV) of ≥ 2.5 were accepted as suspicious lymph nodes.” Though the authors combined anatomical information from CT scans and metabolic data from PET to scans provide a more comprehensive evaluation of lymph node involvement, the cut-off of SUV ≥2.5 was not solid enough to differentiate “node-positive” and “node-negative”. By the way, did all the 109 patients receive PET/CT and CT scans? Otherwise, how much percentage of patients receive PET/CT scan and diagnosed as “node-positive”? This kind of information should be listed.

**Address:** We wholly agreed with you. We reviewed our data to provide suggested information pertaining baseline CT and/or PET/CT scans in line 174-175. **“All 109 patients underwent baseline abdominopelvic and chest CT scans; 71 (65%) underwent baseline PET/CT scans. Among those with baseline PET/CT scans, 17 (24%) showed signs of suspicious EALNs.”** We highly appreciate your input on the cut-off value of SUV ≥2.5 being not solid enough to differentiate “node-positive” and “node-negative”; we abided to the criteria followed by board-certified radiologists at our institution to increase uniformity and minimize variability.

3. “This study only included patients who did not have their EALNs surgically removed.” Since all the suspicious lymph nodes were diagnosed according to imaging scan without pathologically confirmed, the accuracy of diagnosis could be challenged, so as the final conclusion that suspicious EALNs was not significantly associated with the poor prognosis of patients with advanced ovarian cancer.

**Address:** We highly appreciate and wholly agree with your comment pertaining the accuracy of diagnosis being challenged with the lack of pathologic confirmation. However, we deemed the uncertainty of not knowing whether or not suspicious EALNs on baseline CT and/or PET/CT images are indeed malignant a realistic and essential factor that needs to be implemented in this trial. The objective of this study was to evaluate the prognosis of preoperatively detected suspicious EALNs, and without knowing its malignancy or lack thereof, discuss whether resection is necessary.

4. As the authors mentioned, the sample size might influence the statistical power of the analysis.

**Address:** We highly appreciate and wholly agree with your comment pertaining our sample size and its influence on statistical power of the analysis. However, at our institution alone, we are currently unable to accumulate enough data. We acknowledge the need for larger prospective cohorts to assess the clinical impact of suspicious EALNs identified preoperatively on CT and/or PET/CT images in advanced ovarian cancer.

Minor points:
5. The authors stated that among the 36 patients who had suspicious EALNs, there were CPLN (n=24), supraclavicular (n=11), internal mammary (n=4), and inguinal lymph nodes (n=1). I was curious that there were no patients with suspicious mediastinal, axillary, or retrocrural lymph nodes?

**Address:** We wholly agreed with you. For further clarification, we have added suggested details pertaining the anatomical position of detected suspicious EALNs in line 172-174. **“Other sites of suspicious EALNs included not only mediastinal (n=18) such as supraclavicular (n=14), subphrenic (n=1) and hilar (n=1), but also internal mammary (n=6), inguinal (n=2) and axillary (n=3) lymph nodes.”**

6. At line 96 and 205, the reference article should be Ref 17?

**Address:** We apologize for the mistake of citing the wrong reference. We have revised the reference and have cited the appropriate reference of [17] at line 133 and 237.

7. “More specifically, 17 (47.2%) “node-positive patients received neoadjuvant chemotherapy (NACT), while only 19 (26%) in the “node-negative” group received NACT (p=0.027).” Did the imbalance of ratio of NACT between “node-positive” and “node-negative” groups affect the final results?

**Address:** We highly appreciate your comment pertaining the imbalance of neoadjuvant chemotherapy (NACT) ratio between the “node-positive” and “node-negative” groups possibly affecting the final results. This study aimed to determine whether the presence of suspicious EALNs affect oncologic outcomes. Variables that showed significance or had a p-value of <0.20 after multiple linear regression using the backward elimination technique were selected for inclusion in the multivariate model. To further explore the impact of suspicious EALNs on survival while accounting for other potential compounding factors including NACT, multivariate analysis was performed. Thus, we think that the results of this study reflect the effects of NACT.

8. Why was “resistance within third line chemotherapy”, not platinum-resistance in the first or second line, utilized as a factor to do the analysis?

**Address:** We wholly agreed with you. The term we inaccurately phrased “resistance within third line chemotherapy” due to our language barrier and limitation in vocabulary, was actually depicting “platinum-resistance in the first or second line”. We have revised our manuscript to read the suggested “platinum-resistance in the first or second line” phrase.
